# Editorial: Interpersonal skills: individual, social, and technological implications

**DOI:** 10.3389/fpsyg.2023.1209508

**Published:** 2023-06-16

**Authors:** Gerard Beenen, Marina Fiori, Shaun Pichler, Ronald Riggio

**Affiliations:** ^1^Department of Management, College of Business and Economics, California State University, Fullerton, CA, United States; ^2^Department of Research and Development, Swiss Federal University for Vocational Education and Training (SFUVET), Renens, Switzerland; ^3^Department of Psychological Science, Claremont McKenna College, Claremont, CA, United States

**Keywords:** interpersonal skills, social performance, interpersonal mimicry, social relationships, interpersonal dynamics, interpersonal effectiveness

## Introduction

Although interpersonal skills are the touchstone of human social interaction, with hundreds of such skills identified, defining them has remained elusive. Yet, we know interpersonal skills when we see them in action all around us. Students who engage one another to support learning, work colleagues who collaborate productively, supervisors who mentor subordinates in ways that yield satisfaction and task performance, relationships between service providers and customers based on trust and mutual respect—in all these domains, interpersonal dynamics contribute to fulfilling social interactions between individuals and groups. Indeed, productive and meaningful social exchanges are a fundamental need that strikes at the core of what it means to be human (Huston and Burgess, 1979; Ryan and Deci, 2000; Erdogan and Liden, 2002). Our collective experiences of social isolation during the pandemic further underscores that interpersonal skills in everyday life are more important than ever. Therefore, so is research on this topic.

We bring together a collection of six articles that directly or indirectly explore interpersonal skills and dynamics in a variety of domains including educational settings, work settings, and customer service settings. Across these studies, interpersonal skills or interpersonal dynamics are investigated as antecedents, outcomes, mediators and moderators.

## Interpersonal dynamics in education

Widespread use of social media has been associated with a number of developmental and mental health challenges for youth and adolescents (Twenge et al., 2019; O'Reilly, 2020) that can impair wellbeing even into adulthood. This has implications for developing and sustaining meaningful and healthy social relationships, with later potential impacts on employability at work. Serrano-Pintado et al. address this problem indirectly with an initial program evaluation study of interpersonal skills training for 51 adolescent teens in Spain ranging from 12 to 18 years of age. In their study interpersonal competence (social skills) is core to a structured intervention that aims to help young people reduce social anxiety and interpersonal difficulties. Social skills in this context includes interpersonal behaviors that enable students to express emotions and intentions, integrate criticism in a non-defensive manner, minimize interpersonal conflict, and enjoy mutually satisfying relationships. Their intervention demonstrates significant improvements in terms of anxiety reduction from improved self-assessments of assertiveness, interpersonal relationships, and confidence in public speaking. Their findings hold promise that such interventions and training may provide needed relief from the social skill deficits that may be associated with our social media obsessed culture.

Study-abroad experiences represent another educational context where interpersonal skills may be critical for adjustment and wellbeing in an otherwise novel and anxiety-inducing environment—residing in a foreign country where students may lack appropriate communication skills. Khukhlaev et al. investigate 337 international students studying in Russian universities to explore the predictive role of interpersonal mindfulness on intercultural communication effectiveness. In their study, interpersonal mindfulness is a metacognitive antecedent that includes self- and other-awareness in terms of emotions and emotional regulation, as well as intentions and sensitivity pertaining to nonverbal cues in communication. Specifically, they investigate interpersonal dynamics as a motivational frame (i.e., a mindset) to predict effective study abroad experiences. Consistent with anxiety uncertainty management theory, they found that reduced intergroup anxiety mediated the relationship between interpersonal mindfulness and intercultural communication effectiveness. This underscores the importance of a mindset that is attuned to interpersonal and social cues as a prerequisite for students who may aspire to study abroad, yet are naïve of the potential anxiety-inducing effects of such a novel experience.

## Interpersonal dynamics at work

Interpersonal skills play a critical role in the domain of work, both in peer-to-peer relationships and in the supervisor-subordinate dyad (Beenen et al., 2021). Zhang et al. explore the impact of peer-to-peer feedback seeking behaviors on both task performance and workplace wellbeing for a sample of 327 teachers in China. At the core of their model is the quality of relationships between co-workers when seeking feedback. Specifically, robust interpersonal relationships between coworkers mediate the relationship between coworkers' peer-to-peer feedback seeking behavior, and both task performance and wellbeing. On the other hand, these same interpersonal relationships also can serve as a substitute for the benefits of feedback seeking behavior by mitigating (i.e., a negative moderating effect) their impact on performance and wellbeing. In other words, strong interpersonal relationships may be developed through feedback-seeking activities, yet they also serve to diminish the benefits of feedback seeking. This strikes at the core of how strong interpersonal relationships at work forge bonds of mutuality and reciprocity that may yield both enjoyment and performance at work.

The supervisor-subordinate dyad is critical, especially during the period of organizational entry known as newcomer socialization (Bauer et al., 2007). Of central importance in the supervisor-subordinate relationship is the quality of interpersonal exchanges. Deng et al. concentrate on interpersonal dynamics as both a predictor and a mediator. They distinguish supervisor-subordinate dyads focused on occupational skills (newcomer career mentoring), and those focused on interpersonal skills (newcomer psychosocial mentoring). In a study of 157 newcomers and 88 supervisors, consistent with social cognitive career theory, they found psychosocial mentoring predicted social integration and job satisfaction, while career mentoring predicted task mastery, performance and job satisfaction. These relationships were mediated respectively by newcomer social and occupational self-efficacy, and amplified by the learning adaptability of the newcomer. Their findings further reinforce the importance of high-quality interpersonal exchanges between subordinates and supervisors, especially when it comes to onboarding newcomers.

## Interpersonal dynamics of mimicry in the customer and service-provider relationship

Mimicry and social learning represent an instinctive interpersonal dynamic that emerges at the dyadic level whereby the mimicker may convey an interpersonal connection to the mimickee. In the context of a customer and service-provider relationship, the later may serve in a mimicker role and the former in that of the mimickee. Kulesza et al. investigate a “chameleon effect” in this dyad whereby mimicry may facilitate service quality benefits for the customer and reputational benefits for the service provider. In two field experiments (*n* = 46 and *n* = 120), verbal mimicry by the service provider was associated with higher customer satisfaction and provider reputational indicators when compared to a control condition without mimicry. Importantly, the beneficial effects of mimicry spread over to the product and the whole organization, highlighting the marketing implications of the studies' findings. In interpersonal exchanges, mimicry may signal empathy or mockery. It therefore is critical for the mimickee to understand when the mimicker is sending true vs. false signals of liking and affiliation. Adequate amount and timing of mimicry may provide helpful cues for interpreting the true intentions of the mimicker (Kavanagh and Winkielman, 2016). The contextual cues and emotional states associated with both the displays and interpretations of mimicry are an interesting topic for future research.

## Reflecting on the nature of social performance

Our final and most recent entry on this topic takes a step back to reflect on the nature of “social performance” as a quantitative effectiveness indicator of interpersonal exchange. In a review of the literature on social performance as a quantitative outcome, Wild et al. challenge the dominant paradigm of conceptualizing social performance as an analog to intellectual performance. After reviewing mainstream approaches to the assessment of interpersonal skills and social performance, and their related shortcomings, the authors propose a reconceptualization of social performance based on expertise, which integrates, among other factors, individual differences in abilities, skills, traits, and personal experiences relevant for social performance. Aligned with the assumptions of other approaches to expertise, such as in the domain of emotion (Hoemann et al., 2021), the authors recommend multimethod assessments that incorporate complementary features of social expertise and account for the dynamic nature of social performance. Wild et al. conclude by presenting an example of computational modeling in which several individual features (e.g., scores on emotional intelligence, empathy, interpersonal sensitivity) can be considered at the same time and combined into a representative response vector in a multidimensional social performance space. One of the advantages of this approach is it makes it possible to both identify a unique individual or signature profile based on a set of coordinates, as well as to group individuals with similar characteristics (or clusters) based on distance metrics.

## Closing thoughts

Although these Research Topic papers make clear that interpersonal skills are important for effective performance across a variety of contexts, more research is needed as to which types of skills are most important in specific contexts and for specific outcomes. The concept of bandwidth fidelity suggests that the breadth (or specificity) of predictor and criterion should be congruent for stronger validities (Hogan and Holland, 2003). For instance, family-supportive supervision is more strongly related to work-family conflict than general supervisor support (Kossek et al., 2011). Which types of interpersonal skills are more strongly related to in-role performance vs. contextual performance vs. counterproductive work behavior, for instance, and how might this vary across settings? This is an important area for future research on interpersonal skills in organizations.

The healthy and productive interpersonal dynamics investigated in this Research Topic collection are both abounding and lacking all around us. Where they abound, we see students develop and learn, employees thrive, and customers satisfied. Where they are lacking, we see our fellow humans languish, lacking in kindness, empathy and civility, with their needs and goals thwarted. We hope this Research Topic articles inspires more research in this area to facilitate human social performance and thriving.

## Author contributions

GB drafted and edited. MF edited and contributed comments on mimicry and social performance articles. SP provided future research directions. RR edited and provided relevant references. All authors contributed to the article and approved the submitted version.
